# Stauprimide Priming of Human Embryonic Stem Cells toward Definitive Endoderm

**Published:** 2014-02-03

**Authors:** Yaser Tahamtani, Mahnaz Azarnia, Ali Farrokhi, Azadeh Moradmand, Shahab Mirshahvaladi, Nasser Aghdami, Hossein Baharvand

**Affiliations:** 1 Department of Biology, Kharazmi University, Tehran, Iran; 2 Department of Stem Cells and Developmental Biology at Cell Science Research Center, Royan Institute for Stem Cell Biology and Technology, ACECR, Tehran, Iran; 3 Department of Developmental Biology, University of Science and Culture, ACECR, Tehran, Iran

**Keywords:** Definitive Endoderm, Embryonic Stem Cells, Differentiation, Activin A, Stauprimide

## Abstract

**Objective:**

*In vitro* production of a definitive endoderm (DE) is an important issue in stem cell-related differentiation studies and it can assist with the production of more efficient endoderm derivatives for therapeutic applications. Despite tremendous progress in DE differentiation of human embryonic stem cells (hESCs), researchers have yet to discover universal, efficient and cost-effective protocols.

**Materials and Methods:**

In this experimental study, we have treated hESCs with 200 nM of Stauprimide (Spd) for one day followed by activin A (50 ng/ml; A50) for the next three days (Spd- A50). In the positive control group, hESCs were treated with Wnt3a (25 ng/ml) and activin A (100 ng/ml) for the first day followed by activin A for the next three days (100 ng/ml; W/A100-A100).

**Results:**

Gene expression analysis showed up regulation of DE-specific marker genes (*SOX17, FOXA2* and *CXCR4*) comparable to that observed in the positive control group. Expression of the other lineage specific markers did not significantly change (p<0.05). We also obtained the same gene expression results using another hESC line. The use of higher concentrations of Spd (400 and 800 nM) in the Spd-A50 protocol caused an increase in the expression *SOX17* as well as a dramatic increase in mortality rate of the hESCs. A lower concentration of activin A (25 ng/ml) was not able to up regulate the DE-specific marker genes. Then, A50 was replaced by inducers of definitive endoderm; IDE1/2 (IDE1 and IDE2), two previously reported small molecule (SM) inducers of DE, in our protocol (Spd-IDE1/2). This replacement resulted in the up regulation of visceral endoderm (VE) marker (*SOX7*) but not DE-specific markers. Therefore, while the Spd-A50 protocol led to DE production, we have shown that IDE1/2 could not fully replace activin A in DE induction of hESCs.

**Conclusion:**

These findings can assist with the design of more efficient chemically-defined protocols for DE induction of hESCs and lead to a better understanding of the different signaling networks that are involved in DE differentiation of hESCs.

## Introduction

Definitive endoderm (DE) is the origin of all embryonic endodermal organs such as the thyroid, lungs, liver, pancreas and intestines (1-5). Endodermal organs are subject to a number of illnesses, including type 1 diabetes mellitus and liver diseases, for which cell replacement therapies are a promising cure (6-8). Human embryonic stem cells (hESCs) are considered a valuable cell source for these therapeutic applications because of their ability to self-renew and potential to differentiate into different cell types (9). Since these stem cells mimic the *in vivo* developmental events during differentiation, the knowledge of embryology has been used to develop different stepwise protocols to produce endodermal tissues from hESCs (10- 12). The first step in these directed differentiation protocols is the induction of hESCs into DE.

Studies on amniote gastrulation show that the epiblast cells which pass through the anterior primitive streak encounter various concentrations of nodal, a member of the transforming growth factor-beta family (TGF-β) and form mesoderm, in addition to DE (13, 14). Other *in vitro* studies indicate that WNT, phosphatidylinositol 3-kinase (PI3K) and bone morphogenic proteins (BMPs) are important signaling pathways during the DE induction of embryonic stem cells (ESCs) (10, 15-17). The main growth factor inducer in DE differentiation protocols is activin A, which is also a member of the TGF-β family and a replacement for nodal. For example, it has been shown that the use of Wnt3a and activin A induces up to 80% of hESCs to express DE-specific markers such as *SOX17* (15).

During recent years, as an alternative for growth factor inducers, cell-permeable bioactive small molecules (SMs) have been introduced as a means to manipulate stem cell signaling pathways (18-20). SMs can modulate DNA, RNA and protein functions. Their modulatory functions are specific, rapid and reversible. Additionally, they are less expensive (21). SMs are able to efficiently induce ESCs into different cell fates such as neural cells (22, 23), cardiomyocytes (24) and pancreatic cells (23). Inducers of definitive endoderm; IDE1/2 (IDE1 and IDE2), two SM inducers of DE formation, have the capability to efficiently produce DE cells from ESCs (25). SMs also can be used as suppressors of pluripotency in ESCs (21). For example, a 20000 SM screening study has shown that a SM named Stauprimide (Spd) can suppress pluripotency by inhibiting cellular myelocytomatosis oncogene (c-MYC) signaling. This suppression primes ESCs for lineage-specific differentiation (26).

During our previous study (27), we found that Rapamycin priming before activin A induction could efficiently differentiate hESCs into DE. We also observed high expression levels of *SOX17* and *FOXA2* in the hESCs which were primed with Spd before activin induction. Therefore, in this study we further tested the priming capability of Spd and its different concentrations toward activin-induced DE differentiation. We used Spd (200 nM) for the first day and activin A (50 ng/ml) for the following three days (Spd-A50) and after that, we attempted to replace activin A with IDE1/2. Our study showed that treatment of hESCs with Spd- A50 lead to endodermal differentiation. However activin A could not be replaced by SM IDE1/2.

## Materials and Methods

### Human embryonic stem cells culture

Royan H6 (passages 30-40) hESC (28) and Royan H5 (passages 25-30) hESC lines (from Royan Stem Cell Bank,Iran) were used in this experimental study. hESCs were maintained on Matrigel (Sigma-Aldrich, E1270, USA) in hESC medium that consisted of Dulbecco’s modified Eagle’s/Ham’s F12 medium (DMEM/F12, Invitrogen, USA, 21331-020); 20% (v/v) knockout serum replacement (KOSR, Invitrogen, USA, 10828-028); 1% (v/v) non-essential amino acids (Invitrogen, USA, 11140-050); penicillin/ streptomycin (Invitrogen, USA, 15140-122); ITS (insulin 1 mg/mL, transferrin 0.55 mg/mL, selenium 0.00067 mg/mL; Invitrogen, USA, 41400-045); 2 mM L-glutamine (Invitrogen, USA, 25030-032); 0.1 mM B-mercaptoethanol (2 ME, Sigma-Aldrich, USA, M7154); and 100 ng/mL basic fibroblast growth factor (bFGF, Royan Institute, Iran). Cells were grown in 5% CO_2_ at 95% humidity and passaged at a 1:4-1:6 split ratio every seven days with daily media changes.

### Treating hESCs for endoderm formation

**Fig 1:**
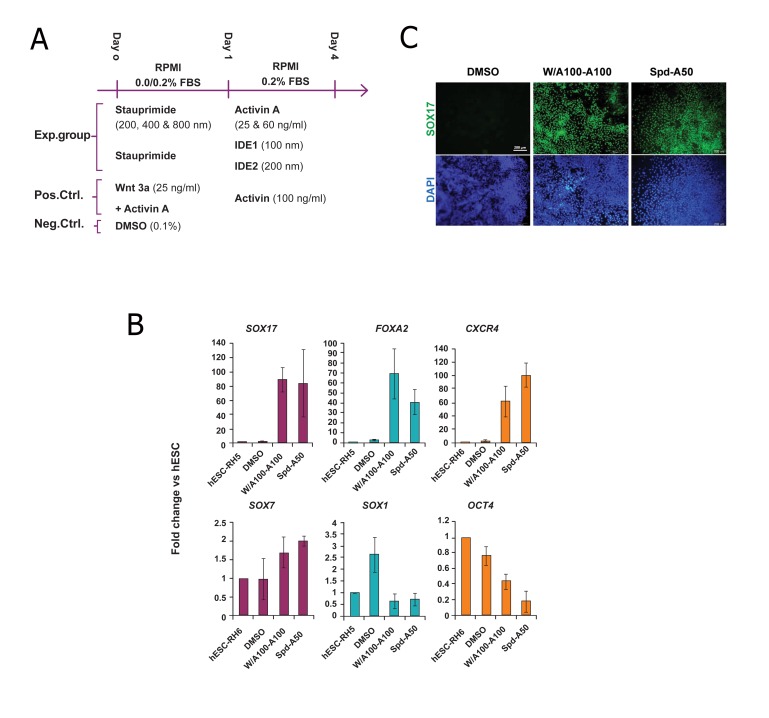
Gene and protein expression analysis of definitive endoderm (DE) markers in experimental and control groups at day 4. A. Diagrammatic representation of the experimental groups (Spd-A50/A25 and Spd-IDE1/2), positive control (W/A100-A100) and negative control (DMSO) for endoderm induction of human embryonic stem cells (hESCs). B. Lineage-specific gene expression analysis of Spd-A50-treated Royan H6 human embryonic stem cells (hESC-RH6). In control groups, hESCs were treated with 0.1% dimethyl sulfoxide (DMSO) for 4 days and considered as the negative control. Cells were treated with Wnt3a and activin A for the first day, followed by treatment with activin A for the next three days (W/A100-A100) as the positive control. As determined by Q-PCR, the DE markers *SOX17, FOXA2* and *CXCR4* highly expressed in Spd-A50-treated Royan H6 hESCs while *SOX7* [visceral endoderm (VE) marker], SOX1 (neuroectoderm marker) and OCT4 (pluripotency marker) had low levels of expression. The target gene expression level was normalized to GAPDH and presented relative to hESC. Data are presented as mean ± SD. C. Immunofluorescent staining of Spd-A50-treated human embryonic stem cells (hESCs) showed *SOX17+* (green) populations comparable to that of W/A100-A100- (positive control) cells. DAPI; 4=,6-diamidino-2-phenylindole.

Before each differentiation step, cultured cells were given a brief wash in Dulbecco’s Phosphate-Buffered Saline with calcium and magnesium (DPBS, Gibco, 104040-182, USA). During differentiation (Fig 1A), 80% confluent hESCs were treated for one day with 200 nM Spd (Santa Cruz, USA, sc-202346) and for next three days with the 50 ng/ml activin A (R&D Systems, 338-AC) or 100/200 nM IDE1/IDE2 (Stemgent, USA, 04-0026 & 04-0027) in RPMI 1640 (Invitrogen, USA, 51800-035) supplemented with nonessential amino acids, L-glutamine, penicillin/ streptomycin, and 0.2% defined fetal bovine serum (FBS, HyClone, SH3007002, USA). For the positive control, as reported previously (29), hESCs were treated with 100 ng/ml activin A and 25 ng/ml Wnt3a (R&D Systems, USA, 5036-WN) in RPMI without FBS for one day followed by 100 ng/ml activin A in RPMI that contained 0.2% FBS for three days. As a negative control, cells were treated with 0.1% DMSO (Sigma-Aldrich, USA, D2650) for four days.

### RNA isolation and real time RT-PCR

Total RNA from samples of the hES, DE, PP, PE cells and hepatocytes were extracted using an RNeasy mini kit (Qiagen, Germany, 74104) according to the manufacturer’s protocol. RNA samples were treated with the RNase-free DNase Set (Qiagen, Germany, 79254) to remove contaminating genomic DNA. The total RNAs were reverse transcribed by the RevertAid H Minus First Strand cDNA Synthesis Kit (Fermentas, USA, K1632) using 0.2 μg of random hexamer primer and 1 μg of total RNA according to the manufacturer’s instructions.

Real time RT-PCR was performed in a Rotor gene 6000 instrument (Corbett Life Sciences) using the following program. Stage 1:95˚C for 10 minutes, stage 2:95˚C for 10 seconds, 60˚C for 30 seconds and 72˚C for 30 seconds, for a total of 40 cycles. The PCR mix in each well included 10 μl of SYBR®Premix Ex Taq™ II (RR081Q, Takara Bio. Inc.), 6 μl of dH2O, 1 μl each of the forward and reverse primers (5 pmol/μl), and 2 μl of single strand cDNA (16 ng/μl) in a final reaction volume of 20 μl. Relative gene expression was analyzed using the comparative Ct method, 2−ΔΔCt (30). The output data from Rotor-Gene 6000 analysis software (version 1.7, Corbett Life Science) were transferred to Microsoft Excel for further analysis. For each sample, the relative expression level was calculated by normalization of target genes to GAPDH as a reference gene and then calibrated against day 0 hESCs. Samples were gathered from 3 to 5 independent biological replicates and all reactions were performed in duplicate. The primers used were designed using Perl Primer software (31). Primer sequences, expected product size and Gene Bank accession numbers are listed in table 1.

### Immunoflourescent staining

For immunofluorescent staining, cells were fixed in 4% w/v paraformaldehyde (Sigma-Aldrich, USA, P6148) for 15 minutes, at 25˚C, permeabilized in 0.1% Triton X-100 for 10 minutes, at 25˚C, and blocked in 10% secondary antibody host serum in 0.5% BSA for 1 hour, at 37˚C, after which they were incubated for 24 hours with goat anti-human *SOX17* antibody (R&D Systems, USA, AF1924) that was diluted 1:100 in 0.5% BSA at 4˚C. Cells were subsequently washed with PBS-Tween 20 (PBST) and incubated with diluted (1:400) rabbit anti-goat IgG-FITC antibody (Sigma-Aldrich, USA, F7367) for 1 hour at 25˚C followed by DNA staining with 4’,6-diamidino- 2-phenylindole (DAPI) (Sigma-Aldrich, USA, D8417) for 3 minutes. Cells were observed under a fluorescence microscope (Olympus, BX51, Japan) and imaged with an Olympus DP72 digital camera mounted to the fluorescent microscope. For negative controls, primary antibodies were omitted and the same staining procedure was followed.

**Table 1:** Primer sequences, annealing temperature and lengths of the amplified products.


Genes	Gene bank code	Forward primer (5' – 3')	Reverse primer (5' – 3')	Annealingtemp (˚C)	Productsize (bp)

GAPDH	NM_002046.3	CTCATTTCCTGGTATGACAACGA	CTTCCTCTTGTGCTCTTGCT	60	121
ACTB	NM_001101.3	AGCACAGAGCCTCGCCTT	CACGATGGAGGGGAAGAC	60	163
OCT3\4	NM_001159542.1	GTTCTTCATTCACTAAGGAAGG	CAAGAGCATCATTGAACTTCA	60	101
SOX1	NM_005986.2	GTGTACCCTGGAGTTTCTG	TAGTCTGTGCCTCTAAAGTG	60	88
SOX7	NM_031439.2	ACGCCGAGCTCAGCAAGAT	TCCACGTACGGCCTCTTCTG	60	73
CXCR4	NM_001008540.1	CACCGCATCTGGAGAACCA	GCCCATTTCCTCGGTGTAGTT	60	80
FOXA2	NM_021784.4	ATGCACTCGGCTTCCAGTAT	TGTTGCTCACGGAGGAGTAG	60	89
SOX17	NM_022454.3	CTCTGCCTCCTCCACGAA	CAGAATCCAGACCTGCACAA	60	102


### Statistical analysis

All experiments were conducted in at least three independent biological replicates. Data from real time RT-PCR were expressed as mean ± SD and the differences of the mean were statistically evaluated by SPSS software (version 16) using one-way analysis of variance (ANOVA) followed by Tukey’s HSD and LSD posthoc tests. P values less than 0.05 were considered significant.

## Results

### Treating Royan H6 hESCs with Spd prior to activin induction

Suppression of regulators of a pluripotency state can lead to the priming of ESCs for differentiation into different lineages (26, 27, 32). Treating hESCs with Spd has been shown to lead to the down regulation of c-MYC which is known as a main factor in self-renewal of hESCs. As shown in figure 1A, we treated Royan H6 hESCs with 200 nM Spd for one day followed by 50 ng/ml activin A for the next three days (Spd-A50). We used a previously reported protocol (W/A100-A100) (11) as the positive control and DMSO treatment as the negative control.

To evaluate the efficiency of our experimental groups for endoderm induction, by the fourth day, we analyzed the cells for expressions of the following DE markers: *SOX17, FOXA2* and *CXCR4* (33, 34); and *SOX7* [visceral endoderm (VE) marker], SOX1 (neuroectoderm marker) and OCT4 (pluripotency marker) by QPCR (Fig 1B).

*SOX17, FOXA2* and *CXCR4* expression levels of Spd-A50-treated hESCs compared with the positive control (W/A100-A100-treated cells) revealed that the Spd-A50 regimen lead to the upregulation of DE marker genes at levels comparable to W/A100-A100. However, the levels of *SOX7* and SOX1 expression remained unchanged and OCT4 down regulated during hESCs treatment with both Spd-A50 and W/ A100-A100 (Fig 1B).

To corroborate the *SOX17* gene expression results in Spd-A50-treated cells, we investigated *SOX17* protein expression. *SOX17+* populations were detected by immunofluorescent staining in the cultures treated with the Spd-A50 protocol (Fig 1C). Similar populations were also detected in the cultures treated with W/A100- A100. Therefore, Spd-A50 protocol was able to upregulate DE-specific markers in hESCs and produce DE cells.

### Effects of Spd and activin concentrations on the efficiency of Spd-A50 protocol

**Fig 2:**
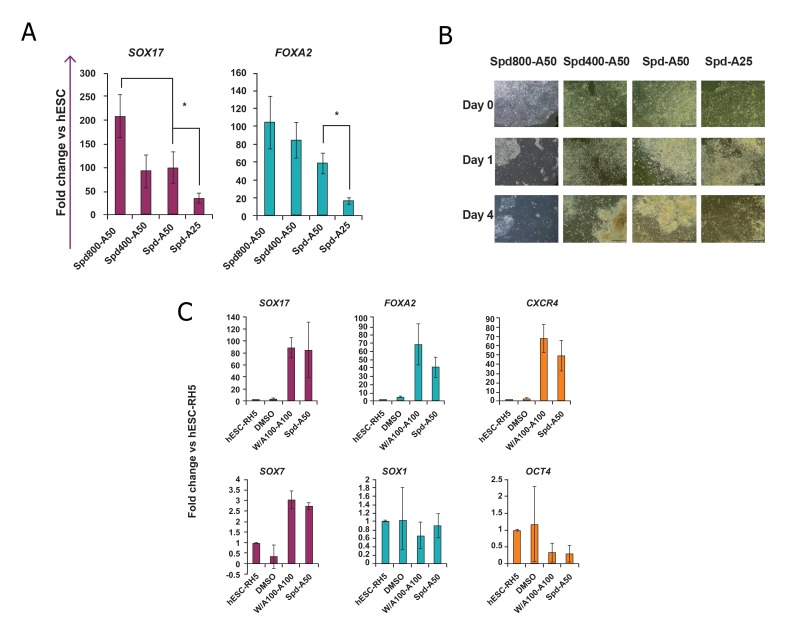
The effects of stauprimide (Spd) and activin A (A) concentrations and human embryonic stem cell (hESC) line on the efficiency of the Spd-A50 protocol. A. hESC-Rh6 were treated with Spd200/400/800-A50 and the Spd-A50 regimen. Samples were analyzed for expressions of *SOX17* and *FOXA2* by Q-PCR at the end of the fourth day. *; Significant difference from all other groups, at least p<0.05 as determined by ANOVA with Tukey’s LSD test. n=3-4. B. Phase contrast images were taken at day 0 (hESC colonies at the beginning of the experiment), day 1 (the same colony, one day after treatment with Spd) and at day 4 (3 days after treatment with activin A). High mortality of the cells was observed after treatment with 800 nM Spd (Spd800-A50 group). C. Lineage-specific gene expression analysis of Spd-A50-treated Royan H5 human embryonic stem cells (hESC-RH5). In control groups, hESCs were treated with 0.1% dimethyl sulfoxide (DMSO) for 4 days and considered as the negative control. Cells were treated with Wnt3a and activin A for the first day followed by activin A for the next three days (W/A100-A100) as the positive control. As determined by Q-PCR, the DE markers *SOX17, FOXA2* and *CXCR4* highly expressed in Spd-A50-treated Royan H5 hESCs while *SOX7* [visceral endoderm (VE) marker], SOX1 (neuroectoderm marker) and OCT4 (pluripotency marker) exhibited low levels of expression. This expression pattern was similar to that obtained from the hESC-RH6 line. The target gene expression level was normalized to GAPDH and presented relative to hESC. Data are presented as mean ± SD.

The primary concentration of Spd (200 nM) was replaced by higher concentrations (400 and 800 nM) of Spd in the Spd-A50 protocol. Treated hESCs were investigated for the expressions of DE markers *SOX17* and *FOXA2* by Q-PCR. Although the levels of DE marker genes in Spd800-A50-treated hESCs were significantly (p<0.05) higher than Spd-A50-treated cells (Fig 2A), phase contrast images showed that treating hESCs with 800 nM Spd lead to a very high cell mortality (Fig 2B). As the *SOX17* and *FOXA2* expression levels were not significantly different in Spd 400 and Spd-A50-treated cells, the 200 nM concentration of Spd was selected for the remainder of the experiments. Additionally, the lower concentration of activin A (25 ng/ml) was tested (Spd-A25). According to Q-PCR results, this concentration of activin A caused a significant decrease in the expression levels of DE marker genes.

The Spd-A50 protocol was tested on another hESC line (Royan H5) and the same gene expression results as hESC-RH6 were obtained (Fig 2C).

### Effects of Spd and activin concentrations on the efficiency of Spd-A50 protocol

**Fig 3:**
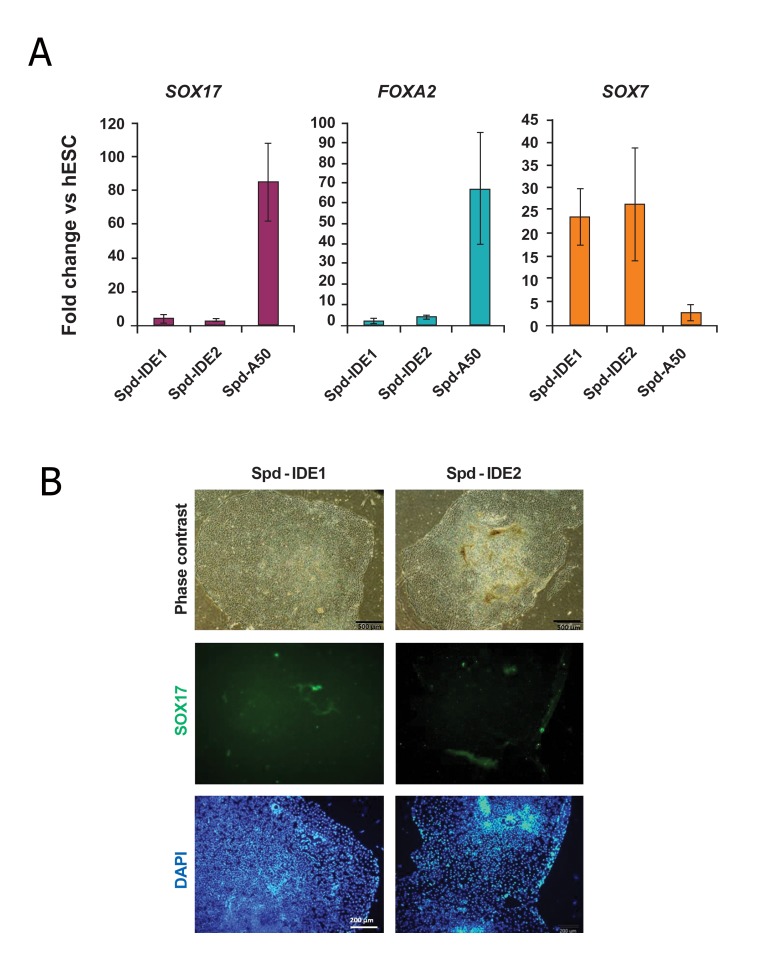
Gene and protein expression analysis of definitive endoderm (DE) and visceral endoderm (VE) markers in human embryonic stem cell (hESCs) treated with Spd-IDEs. A. After treating hESCs with Spd-IDE1/2, samples were analyzed for the expressions of *SOX17, FOXA2* and *SOX7* by Q-PCR at the end of the fourth day. The target gene expression level was normalized to GAPDH and presented relative to hESC. Data are presented as mean ± SD. Asterisk indicates significant difference between the shown groups (p<0.05 as determined by ANOVA with Tukey’s LSD test; n=3). B. Immunofluorescent staining of Spd-IDE1/2-treated hESCs indicated no *SOX17+* cells in the groups. DAPI; 4=,6-diamidino- 2-phenylindole.

It has previously been shown that treatment of hESCs with IDE1/2 induced DE differentiation (25). Thus we replaced activin A with IDE1/2 in the Spd-A50 protocol and treated hESCs with this new regimen (Spd-IDE1/2). As shown in figure 3, this replacement caused a dramatic decrease in *SOX17* (also at the protein level) and *FOXA2* expression. However, the VE marker, *SOX7* up regulated in cells treated with IDE1/2.

## Discussion

For DE differentiation, we used a twostep protocol. In the first step, hESCs were treated with Spd, a previously introduced SM suppressor of pluripotency (26, 27) and in the second step they were exposed to activin A, an inducer of endoderm formation. We showed that Spd-A50 regimen lead to upregulation of DE-specific markers (*SOX17, FOXA2* and *CXCR4*) while no increase was observed in VE-specific gene (*SOX7*) expression. There was no difference in the DE-specific gene expression patterns between Spd- A50- and W/A100-A100-induced DE cells. As reported before, Spd has been shown to inhibit nuclear localization of nucleoside diphosphate kinase B (DNPK B), which led to the down regulation of c-MYC a known main factor in hESC self-renewal. Thus it could prime hESCs for differentiation (26). As we previously reported (27), pre-treatment of hESCs with SM suppressors of pluripotency could be a strategy to improve the efficiency of DE induction.

Activin/Smad2/3 is an important signaling pathway during endoderm formation (15, 35) and widely used in DE differentiation protocols. We reduced the concentration of activin A to 25 ng/ml in the Spd-A50 protocol, which led to a dramatic reduction in the expression of DE-specific genes. This agreed with previous reports that different concentrations of growth factor activin A have different inducing effects on hESCs (3).

IDE1 and IDE2 have been previously reported to be inducers of DE. While the exact mechanisms of their action are not known, the activation of the TGF-β signaling pathway has been shown in hESCs treated with these SMs(25). In contrast, we did not observe any up regulation of DE markers in Spd-IDE1/2-treated hESCs while the VE marker gene (*SOX7*) showed a significant up regulation in these cells. This was possibly due to the different abilities of hESC lines for differentiation under identical conditions as reported in numerous studies (36).

## Conclusion

This study showed that while the Spd-A50 protocol could lead to efficient DE differentiation, replacement of activin A with two previously reported inducers of DE (IDE1/2) dramatically reduced the efficiency of DE differentiation in the tested hESC lines.
